# Adaptive Bit-Labeling Design for Probabilistic Shaping Based on Residual Source Redundancy

**DOI:** 10.3390/e25040586

**Published:** 2023-03-29

**Authors:** Chen Chen, Qiwang Chen, Sanya Liu, Lin Zhou

**Affiliations:** Xiamen Key Laboratory of Mobile Multimedia Communications, College of Information Science and Engineering, Huaqiao University, Xiamen 361021, China; chen_chen@hqu.edu.cn (C.C.);

**Keywords:** bit labeling, joint source-channel coded modulation, quadrature amplitude modulation (QAM), protograph LDPC codes

## Abstract

By using the residual source redundancy to achieve the shaping gain, a joint source-channel coded modulation (JSCCM) system has been proposed as a new solution for probabilistic amplitude shaping (PAS). However, the source and channel codes in the JSCCM system should be designed specifically for a given source probability to ensure optimal PAS performance, which is undesirable for systems with dynamically changing source probabilities. In this paper, we propose a new shaping scheme by optimizing the bit-labeling of the JSCCM system. Instead of the conventional fixed labeling, the proposed bit-labelings are adaptively designed according to the source probability and the source code. Since it is simple to switch between different labelings according to the source probability and the source code, the proposed design can be considered as a promising low complexity alternative to obtain the shaping gain for sources with different probabilities. Numerical results show that the proposed bit-labelings can significantly improve the bit-error rate (BER) performance of the JSCCM system.

## 1. Introduction

Bit interleaved coded modulation (BICM) [1] with uniformly distributed symbols leads to a shaping loss and prevents the performance from approaching the Shannon limit for higher order modulation [2]. To close the gap to the Shannon limit, probabilistic amplitude shaping (PAS) schemes were introduced using a distribution matcher (DM) [3]. In [4,5], the PAS schemes using non-binary and protograph low-density parity-check (LDPC) codes were investigated for BICM systems. To further reduce the complexity of binary DM, a PAS scheme via simplified sign-bit shaping was proposed for high spectral-efficiency coding in [6]. In [7], a probabilistic shaping scheme for nongray labelled QAM constellations was proposed.

Although the PAS schemes for BICM have been extensively investigated in recent years, most of them only focus on uniform inputs. Specifically, the constant composition distribution matching (CCDM) utilized in the standard PAS works can only transform Bernoulli (1/2) distributed input bits into output symbols [8].

However, in realistic applications, natural sources often contain substantial amounts of redundancy due to the non-uniform distribution of the source symbols and the source memory. In such cases, source codes should be utilized, which can be divided into two categories: variable-length codes (VLCs) and fixed-length codes (FLCs). Although VLCs exhibit high compression rates, a few bit errors after the channel decoder may dramatically corrupt the decoded source data. To prevent this catastrophic error propagation, FLCs have been proposed and successfully demonstrated for several applications such as GSM and wide-band adaptive multi-rate (WB-AMR) speech transmission [9,10] and image/video transmission [11,12,13]. Because of the limit of the length, the FLCs still exhibit residual redundancy in their outputs.

For non-uniform sources, in [14], protograph LDPC codes are optimized under binary modulation with unequal power allocation. By using the source residual redundancy after source coding to achieve probabilistic shaping, a joint source-channel coded modulation (JSCCM) system was proposed [15]. In [15], the design of the source code parameter is targeted specifically at a given source probability, and thus the residual redundancy left after source coding can be properly used to shape the transmitted symbols. Accordingly, the source-channel code pairs optimized at one source probability may lead to extremely poor performance at another source probability due to the mismatch of the source code parameter and the source probability. However, this characteristic is undesirable for some systems with varying levels of data redundancy since the source-channel code pairs should be optimized at different source probabilities to ensure the PAS performance.

In this paper, a low complexity method to obtain the shaping gain for sources with different probabilities is proposed for the JSCCM system. The main contributions of this paper are as follows:

- By studying the effects of bit-labeling in JSCCM systems, it is found that good bit-labelings for different source codes or different source probabilities could be different.
- Based on the achievable system rate analysis, a new shaping scheme for the JSCCM system is proposed by optimizing the bit-labeling.
- In contrast to the fixed Gray labeling [16], the adaptive design of bit-labelings for the JSCCM system is proposed according to the source codes and the source probabilities. Since it is much simpler to switch between labelings than to optimize the source-channel code pairs for different source probabilities, it is attractive for systems with changing source statistics.

The remainder of the paper is organized as follows. Section 2 presents the JSCCM system model. Section 3 proposes an adaptive design algorithm of the bit-labeling. Section 4 discusses the performance of the system using the adaptively designed labelings in comparison with the fixed labeling. Finally, Section 5 concludes the paper.

## 2. System Model

**Figure 1:**
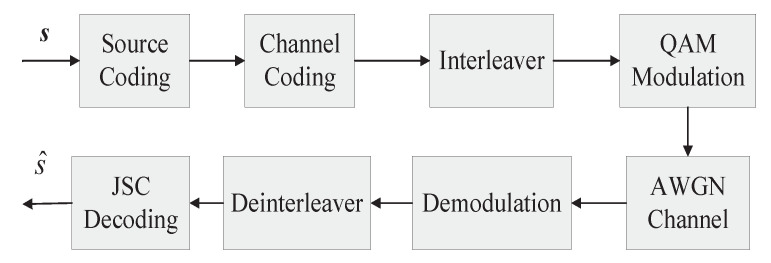
The structure of a JSCCM system.

The structure of the JSCCM system over AWGN channels is shown in Figure 1. A non-uniform memoryless binary (“0” and “1”) source is considered in this paper, where the probability of “1” is represented as *p* (p≠0.5). The source entropy is therefore given by

(1) $$H\left(p\right)=-p\log_{2}p-\left(1-p\right)\log_{2}\left(1-p\right)<1.$$

Let us consider source protograph LDPC codes with base matrix $B_{s}$ and channel protograph LDPC codes with base matrix $B_{c}$. Then, the progressive edge growth (PEG) algorithm is employed to generate the corresponding low-density matrices $H_{s}$ and $H_{c}$ by the copy-and-permute operation [17].

The encoding process of the JSCCM system comprises two steps as follows. Firstly, the source bit sequence s is compressed by using the source code as

(2) $$b^{T}=H_{s}s^{T},$$

where b is the compressed bit sequence. Then, the compressed bit sequence is protected by the channel code. For systematic binary channel encoding, a systematic generator matrix can be constructed from the parity-check matrix $H_{c}$ and is represented by

(3) $$G_{c}=\left[P|I\right],$$

where I is the identity matrix. The systematic channel codeword x is thus obtained as

(4) $$x=bG_{c}=\left[c|b\right],$$

where c=bP is the parity bit sequence.

Assume a quadrature amplitude (QAM) alphabet with $2^{m}$ signal points, where *m* is the modulation order. By setting the channel code rate to be (m−1)/m, the channel codeword x can be composed of a parity bit sequence c of length *N* and a compressed bit sequence b of length N×(m−1), where *N* represents the lifting factor. Thus, the sequence x is organized as a N×m binary matrix by the interleaver as

(5) $$\begin{array}{cc} M_{x}^{\left(1\right)}= & \left[\begin{array}{ccccc} c_{1} & b_{1}^{1} & b_{1}^{2} & \cdots & b_{1}^{m-1}\\ c_{2} & b_{2}^{1} & b_{2}^{2} & \cdots & b_{2}^{m-1}\\ \vdots & \vdots & \vdots & \vdots & \vdots \\ \underset{︸}{c_{N}} & \underset{︸}{b_{N}^{1}} & \underset{︸}{b_{N}^{2}} & \cdots & \underset{︸}{b_{N}^{m-1}} \end{array}\right]\\ & \begin{array}{ccccc} \;\begin{array}{c} b^{0} \end{array} & \;b^{1} & \;b^{2} & \;\cdots & \;b^{m-1} \end{array} \end{array},$$

where every column forms a bit level $b^{i}$ for 0≤i≤m−1.

In (5), the parity bits in c are all transferred to the bit level $b^{0}$ and the compressed bits in block $b^{i}$, 1≤i≤m−1 are transferred to the bit level $b^{i}$. Subsequently, the QAM symbol sequence $X=\left(X_{1},X_{2},\cdots ,X_{N}\right)$ for transmission is obtained by row-wise mapping according to the mapping rule.

With the constellation scaling α>0, the AWGN channel is described by the input–output relationship

(6) Y=αX+Z,

where X is the modulated symbol sequence, Y is the received symbol sequence, and Z is the AWGN sequence.

The overall transmission rate of the JSCCM system is defined in “source bits/channel symbol” as

(7) $$R=\frac{mR_{c}}{R_{s}}=\frac{m-1}{R_{s}},$$

where $R_{s}$ and $R_{c}$ represent the source coding rate and the channel coding rate, respectively.

At the receiver, after demodulation and de-interleaving, the joint source and channel decoder is applied to reconstruct the source bit sequence.

## 3. Analysis and Design of Bit-Labeling

As shown in [15], in JSCCM systems, the parity bits transferred to the bit level $b^{0}$ are uniformly distributed. Meanwhile, the compressed bits transferred to the bit level in block $b^{i},1\leq i\leq m-1$ can be computed by (2) as the modulo-2 sum of the source bits. Therefore, the probability distribution of the compressed bits is determined essentially by the row weight distribution of $B_{s}$ in combination with the source probability distribution *p* as [15,18]

(8) pBsi(1)=∑k=0,oddwiwikpk1−pwi−k,i=1,2,⋯,m−1

where $w_{i}$ represents the weight of the *i*-th row of $B_{s}$ and wik denotes the binomial coefficients. Note that $p^{i}\left(1\right)+p^{i}\left(0\right)=1$ and $p^{i}\left(1\right)\neq p^{i}\left(0\right)$ for i=1,2,⋯,m−1 when p≠0.5, thus leading to nonuniform input symbol distributions.

Let X denote a set of $2^{m}$-ary QAM constellation points. After interleaving, a symbol mapper maps an *m*-bit vector $x=\left[b^{0},\cdots ,b^{m-1}\right]\in F_{2}^{m}$ onto X=ϕ(x)∈X, where $\phi \left(\cdot \right):F_{2}^{m}\to X$ is a symbol mapping function. Assume that the bits at the input of the modulator are independent, the symbol probabilities for transmission obtained as follows

(9) $$\begin{array}{c} P_{B_{s}}\left(X,\phi \right)=\frac{1}{2}\prod_{i=1}^{m-1}p^{i}\left(b^{i}\right), \end{array}$$

where $p^{i}\left(u\right)$ represents the probability of transmitting a bit u∈{0,1} at bit position $b^{i}$.

For a given source probability *p* and a source code matrix $B_{s}$, the probability of every bit level can be identified. Then, the symbol probability is determined by the mapping function ϕ(·), which maps each length-*m* binary vector $\left[b^{0},\cdots ,b^{m-1}\right]$ to a corresponding symbol *X*, and thus we explicitly indicate this dependence in (9).

### 3.1. Effects of Bit-Labelings

**Figure 2:**
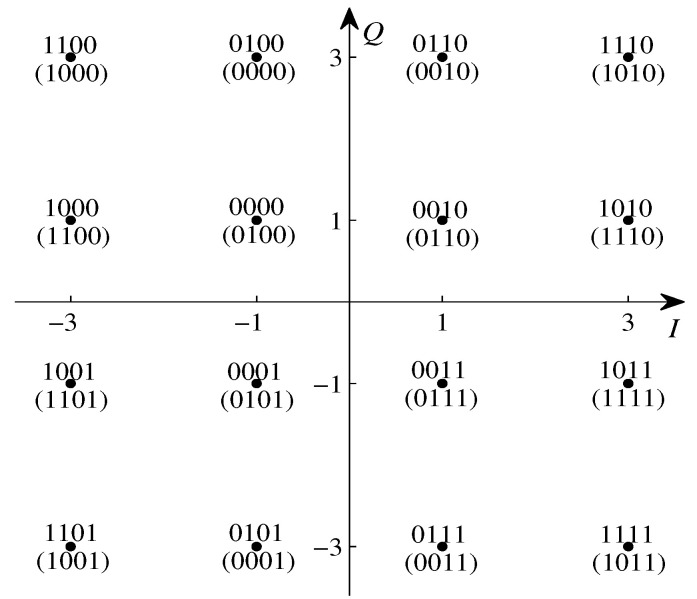
A 16-QAM constellation with two labelings: *L* and $L^{\prime }$(in parentheses).

With an aim to illustrate the effects of bit-labelings on the JSCCM system, we compare the BER performance of two different Gray labelings. Figure 2 shows a 16-QAM constellation with *L* and $L^{\prime }$ labelings, which are both under the rule of one-bit discrepancy between adjacent binary labels.

**Figure 3:**
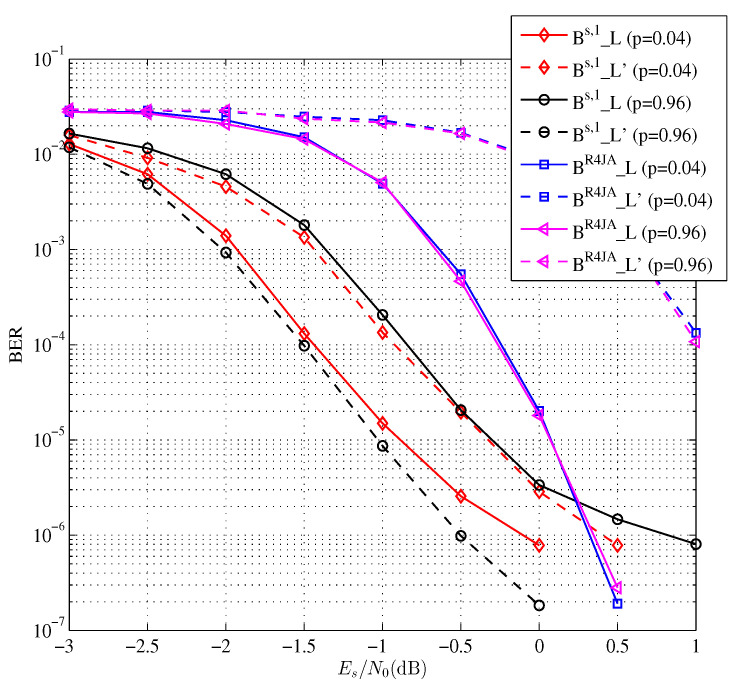
BER performance of JSCCM system with two labelings.

Figure 3 plots the simulated BER results of those two labelings with different source codes ($B^{s,1}$ [15] and $B^{R4JA}$ [19]) and different source probabilities (p=0.04 and p=0.96). Note that the two source probabilities contribute to the same source entropy according to (1). It can be found that the labeling *L* with a competitive advantage at p=0.04 is outperformed by $L^{\prime }$ at p=0.96 when $B^{s,1}$ is utilized. Meanwhile, it can be observed that the better labeling for $B^{s,1}$ results in worse performance for $B^{R4JA}$ when p=0.96. Therefore, the good bit-labelings for different source probabilities or different source codes can be different. An adaptive design of bit-labeling according to the source probability and the source code is essential for the JSCCM system.

### 3.2. An Adaptive Design Scheme of Bit-Labeling

In this section, we consider the adaptive design procedure that optimizes the bit-labeling according to the source probability and the source code for a given target transmission rate by the achievable system rate analysis.

For the reliable transmission of an asymmetric source with entropy H(p) over a memoryless AWGN channel with capacity *C* at a rate of *R* source bits per channel symbol, the Shannon limit can be expressed as [20]

(10) H(p)R<C,

where *C* represents the capacity of two independent Gaussian channels in parallel given by $\log_{2}\left(1+R\cdot E_{s}/N_{0}\right)$, $E_{s}$ is the average energy per source bit, and $N_{0}$ is the one-sided noise power spectral density. Given *p* and *R*, the Shannon limit can be interpreted by the smallest $E_{s}/N_{0}$ satisfying (10) and expressed as

(11) $${\left.\frac{E_{s}}{N_{0}}\right|}_{\mathrm{Shannon}}=\frac{2^{H(p)R}-1}{R}.$$

For the QAM-modulated AWGN channel with the alphabet X and the input probability distribution $P_{B_{s}}\left(X,\phi \right)$ given by (9), the channel capacity is calculated as

(12) $$C_{B_{s}}\left(\frac{E_{s}}{N_{0}},\phi \right)=\sum_{X\in X}P\left(X,\phi \right)\int_{-\infty }^{+\infty }P_{\alpha }\left(Y|X\right)\log_{2}\frac{P_{\alpha }\left(Y|X\right)}{\sum_{X^{\prime }\in X}P\left(X^{\prime },\phi \right)P_{\alpha }\left(Y|X^{\prime }\right)}dY,$$

where $\alpha =\sqrt{\frac{E_{s}}{N_{0}}RN_{0}/\left(\sum_{X\in X}P\left(X,\phi \right){\left|X\right|}^{2}\right)}$ and $P_{\alpha }\left(Y|X\right)=\frac{1}{\pi N_{0}}\exp\left\{-\frac{{\left(Y-\alpha X\right)}^{2}}{N_{0}}\right\}$,

According to (12), $C_{B_{s}}\left(\frac{E_{s}}{N_{0}},\phi \right)$ for a given source probability *p* and a source code matrix $B_{s}$ is conditional on $E_{s}/N_{0}$ and the mapping function ϕ. Thus, by properly designing ϕ, a shaping gain in $E_{s}/N_{0}$ can be obtained. However, it is impossible to derive an analytical solution to ϕ, and the search for ϕ has to resort to an exhaustive method.

In Algorithm 1, an adaptive design procedure is proposed to search for the optimal mapping function ϕ, also known as the optimal bit-labeling, according to the given source probability *p* and the source code $B_{s}$ at a target transmission rate *R*. For a practical search, we start from the smallest $E_{s}/N_{0}$ satisfying (10), denoted by ${\left.\frac{E_{s}}{N_{0}}\right|}_{\mathrm{Shannon}}$, and gradually increase the value of $E_{s}/N_{0}$ until the achievable system rates, denoted as $C_{B_{s}}\left(\frac{E_{s}}{N_{0}},\phi \right)/H\left(p\right)$, is around *R*, at which point we wish to optimize the bit-labeling. Then, we evaluate the channel capacity $C_{B_{s}}\left(\frac{E_{s}}{N_{0}},\phi \right)$ for different mapping functions and select the mapping function which exhibits the highest capacity. Note that the number of different labelings is very large for the constellation with high-order modulations such as 16QAM, so we focus on Gray codes due to their good error rate performance.

**Figure 4:**
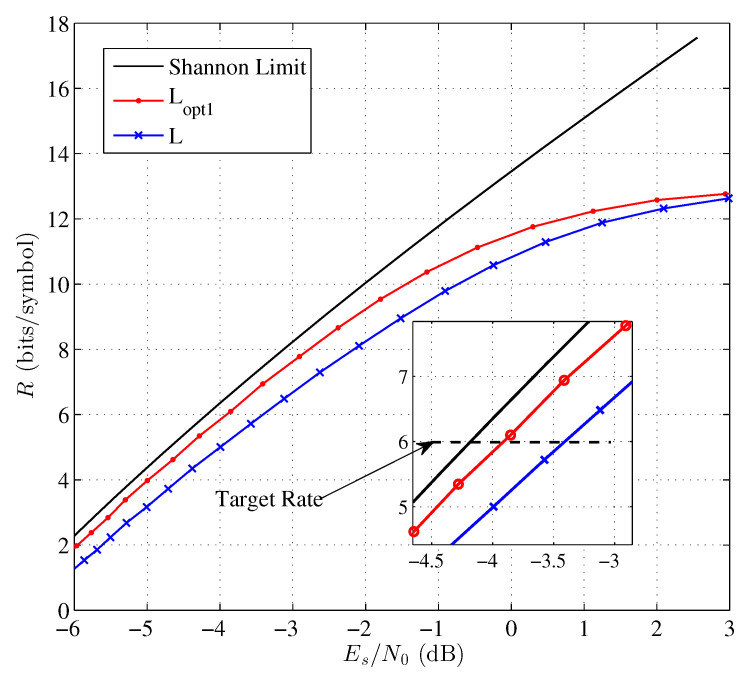
Achievable system rates for a 16QAM constellation using different labelings.

Example: Consider *p* = 0.05, *m* = 4, *R* = 6 bits/symbol, Δ=0.1 dB. The source code is the 1/2-rate-$B^{s,1}$ code [15]. In Figure 4, the achievable system rates $R=C_{B_{s}}\left(\frac{E_{s}}{N_{0}},\phi \right)/H\left(p\right)$ versus $E_{s}/N_{0}$ for a 16QAM constellation using the optimized labeling $L_{opt1}$ and labeling *L* are provided. For comparison, the Shannon limits C/H(p) are also provided. Compared to labeling *L*, when *R* is 6 bits/symbol, the JSCCM system with the optimized labeling can obtain 0.5 dB shaping gain, which reduces the gap to the Shannon limit.
**Algorithm** **1** Adaptive Bit-Labeling Design**Require:** $p,B_{s},R,\Delta ,\phi_{ini}$1:C←H(p)R;2:${\mathrm{snr}}_{\min}\leftarrow 10\log_{10}\left[\left(2^{C}-1\right)/R\right]$;       \∗Shannon limit∗\3:$\mathrm{snr}\leftarrow {\mathrm{snr}}_{\min}$, $\phi \leftarrow \phi_{ini}$4:**for** all gray mappings $\phi^{\prime }$ ($\phi^{\prime }\neq \phi_{ini}$) **do**5:   compute $P_{B_{s}}\left(X,\phi \right)$ with *p*, $B_{s}$ and ϕ;6:   compute $C_{B_{s}}\left(10^{\mathrm{snr}/10},\phi \right)$ with $P_{B_{s}}\left(X,\phi \right)$, snr and ϕ;7:   **while** $C_{B_{s}}\left(10^{\mathrm{snr}/10},\phi \right)/H\left(p\right)<R$ **do**8:     snr=snr+Δ9:   **end while**10:   compute $C_{B_{s}}\left(10^{(\mathrm{snr}-\Delta )/10},\phi^{\prime }\right)$ and $C_{B_{s}}\left(10^{\mathrm{snr}/10},\phi^{\prime }\right)$;11:   **if** $C_{B_{s}}\left(10^{(\mathrm{snr}-\Delta )/10},\phi^{\prime }\right)\geq C_{B_{s}}\left(10^{(\mathrm{snr}-\Delta )/10},\phi \right)$ and $C_{B_{s}}\left(10^{\mathrm{snr}/10},\phi^{\prime }\right)\geq C_{B_{s}}\left(10^{\mathrm{snr}/10},\phi \right)$ **then**12:     $\phi \leftarrow \phi^{\prime }$;13:     $\mathrm{snr}\leftarrow {\mathrm{snr}}_{\min}$14:   **end if**15:**end for**15:**return** ϕ;

To further verify the merits of the proposed bit-labelings, we present some bit-error-rate (BER) results of the JSCCM systems with 16QAM over an AWGN channel for different source codes and source probabilities. For all the cases, the same Protograph LDPC code (3/4-rate-$B_{c}^{U}$ [21]) is employed as a channel code. And four Protograph LDPC codes, including the 1/2-rate-$B^{s,1}$ code [15], the 1/2-rate-$B^{R4JA}$ code [19], the 3/8-rate-$B^{s,2}$ code [22], and the 3/8-rate-$B^{s,3}$ code [23], are used for source coding as follows:

(13) $$B^{s,1}=\left(\begin{array}{cccccc} 1 & 1 & 3 & 3 & 1 & 0\\ 1 & 0 & 5 & 2 & 0 & 1\\ 1 & 2 & 3 & 3 & 1 & 1 \end{array}\right).$$

(14) $$B^{R4JA}=\left(\begin{array}{cccc} 3 & 1 & 1 & 1\\ 1 & 2 & 1 & 2 \end{array}\right).$$

(15) $$B^{s,2}=\left(\begin{array}{cccccccc} 2 & 1 & 1 & 1 & 1 & 1 & 1 & 1\\ 1 & 2 & 0 & 1 & 2 & 1 & 1 & 0\\ 1 & 1 & 2 & 1 & 0 & 2 & 0 & 1 \end{array}\right).$$

(16) $$B^{s,3}=\left(\begin{array}{cccccccc} 2 & 1 & 0 & 2 & 3 & 1 & 1 & 1\\ 1 & 2 & 1 & 2 & 1 & 3 & 0 & 2\\ 1 & 1 & 2 & 1 & 1 & 0 & 3 & 2 \end{array}\right).$$

## 4. Experimental Results

**Figure 5:**
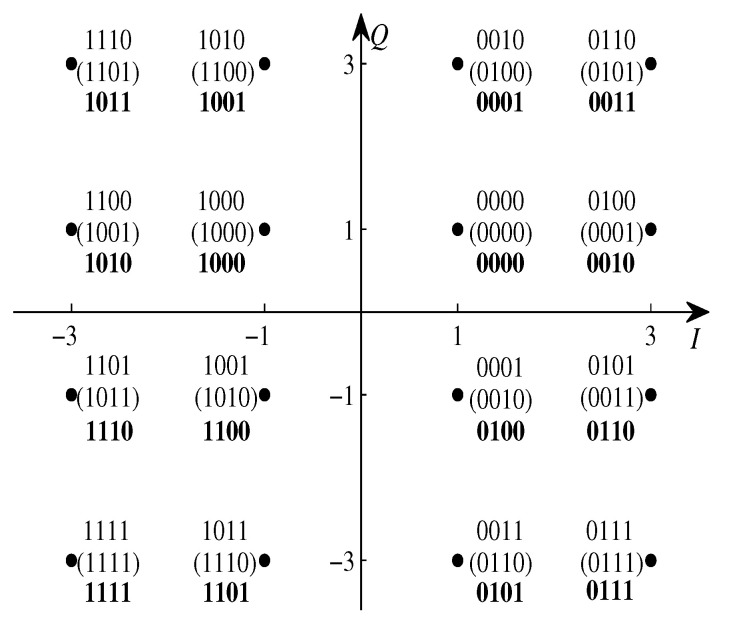
The optimized bit-labelings $L_{opt1}$, $L_{opt2}$ (in parentheses) and $L_{opt3}$ (in bold) for JSCCM system.

**Figure 6:**
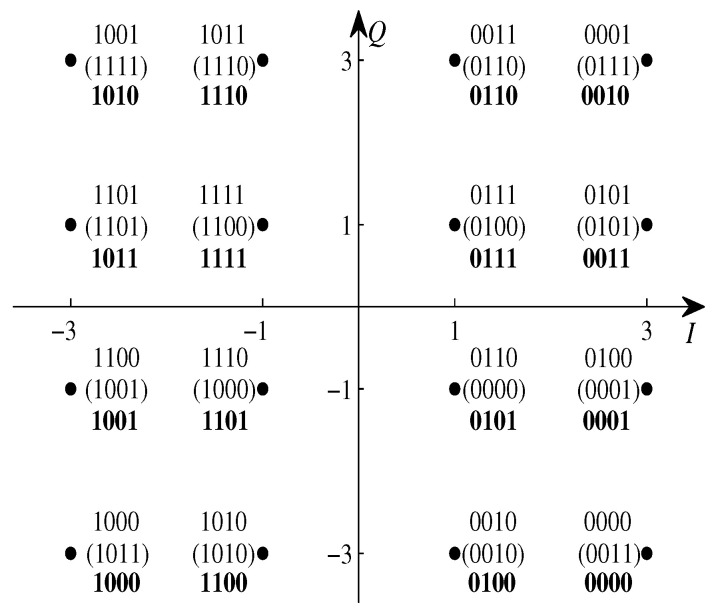
The optimized bit-labelings $L_{opt4}$, $L_{opt5}$ (in parentheses) and $L_{opt6}$ (in bold) for JSCCM system.

The system settings for different cases are presented in Table 1. In particular, since the source coding rates are 1/2 and 3/8, the resulting system rates are derived from (7) are 6 bits and 8 bits per symbol, respectively. In all experiments, the length of the source sequence is set as 3600 bits. For cases with different source coding rates, the source and channel codes are designed from case to case with respect to the source and channel coding rates, respectively. The optimal labelings obtained by Algorithm 1 for different cases are shown in Figure 5 and Figure 6 above the constellation points.

**Figure 7:**
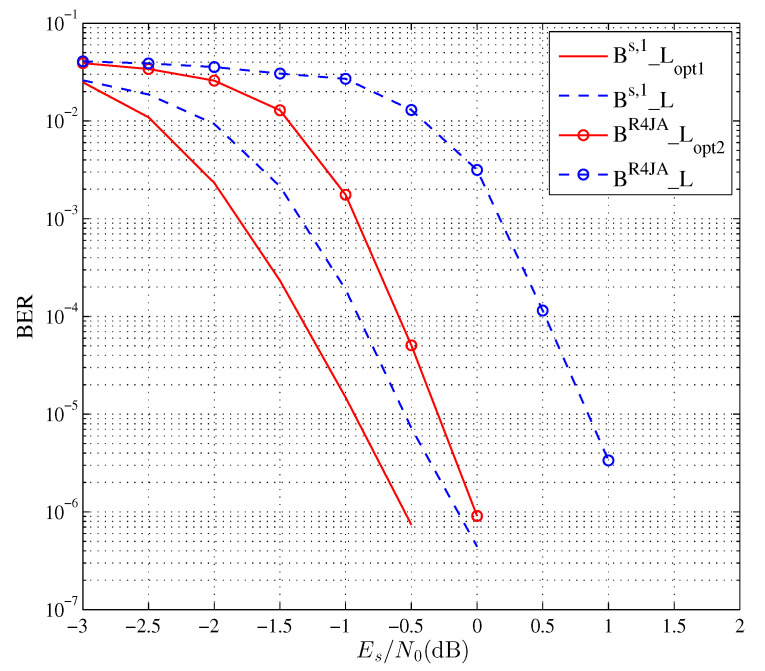
BER comparison of different labelings with p=0.05 and R=6.

**Figure 8:**
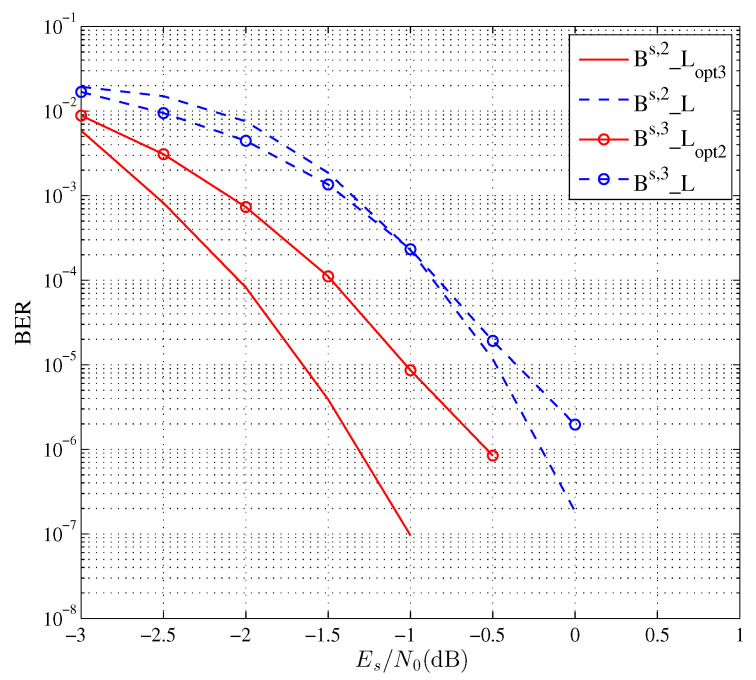
BER comparison of different labelings with p=0.03 and R=8.

**Figure 9:**
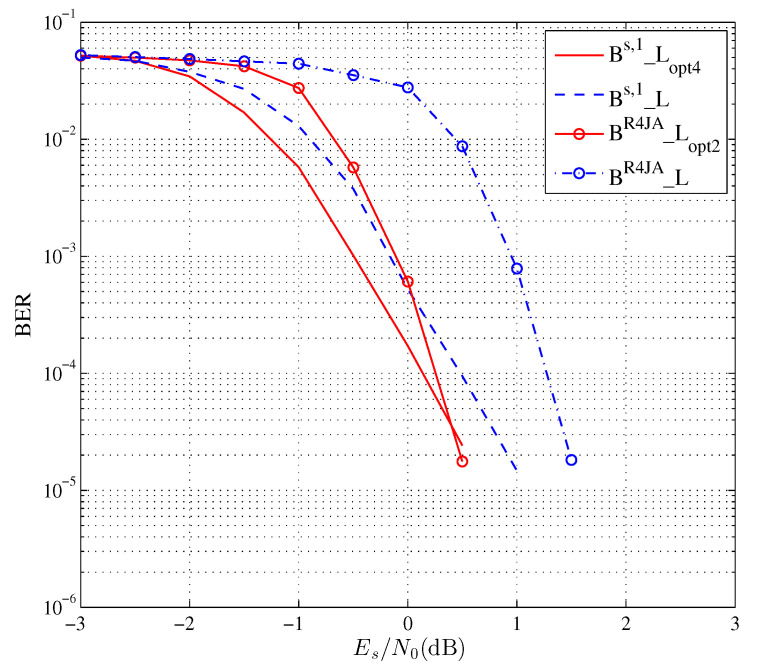
BER comparison of different labelings with p=0.94 and R=6.

**Figure 10:**
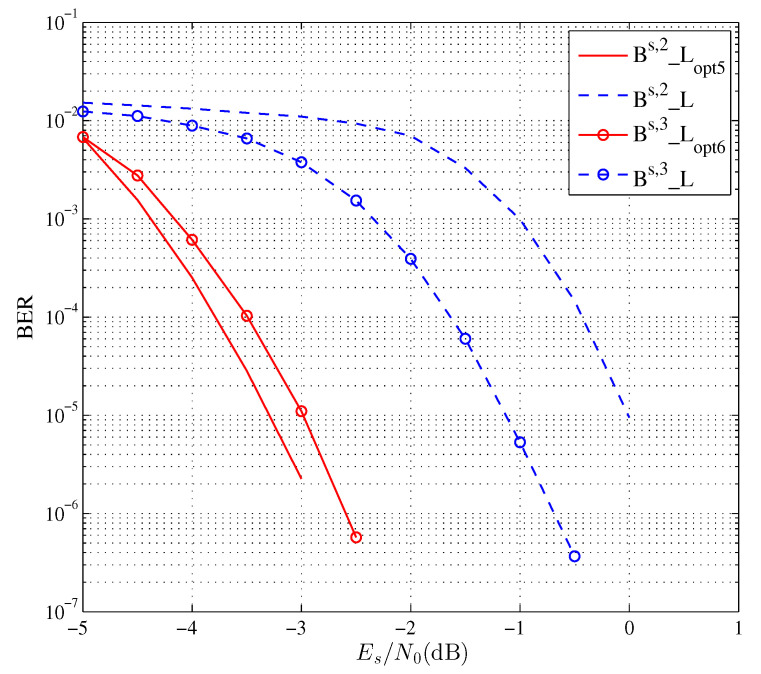
BER comparison of different labelings with p=0.98 and R=8.

The BER curves of the system with the optimized labelings (solid lines) and the labeling *L* (dashed lines) for different cases are depicted in Figure 7, Figure 8, Figure 9 and Figure 10. For instance, in Figure 7, for the source code $B^{s,1}$, the optimized labeling $L_{opt1}$ achieves approximately 0.5 dB gain over labeling *L* at a BER of $10^{-6}$. Additionally, for the source code $B^{R4JA}$, our designed $L_{opt2}$ outperforms *L* by approximately 1.3 dB at a BER of $10^{-5}$. Similar observations can be found in Figure 8, Figure 9 and Figure 10. Particularly, the source code $B^{s,2}$ in Figure 10, which has extremely poor BER performance with labeling *L*, achieves a performance gain of at least 3 dB after labeling design. Specifically, the performance gain varies depending on the matching degree of the utilized labeling to the source code and the source probability. For example, using a bit labeling that is highly mismatched to the source probability and the source code could yield a loss of shaping gain, and the performance can be greatly improved by the proposed design of bit-labeling. If the labeling is well matched to the source probability and the source code, the advantage of our method diminishes.

## 5. Conclusions

In this paper, a new PAS scheme via bit-labeling design for the JSCCM system is proposed. In contrast to the fixed labeling, an algorithm of adaptive bit-labeling design is proposed according to the source probability and the source code based on the achievable system rate analysis. Simulation results demonstrate that the adaptively designed bit-labelings significantly improve the BER performance of the JSCCM system.

## Figures and Tables

**Table 1:** System settings for performance comparisons.

Source Probability	Source Code	Target Rate (Bits/Symbol)	Optimized Labeling
p=0.05	$B^{s,1}$	6	$L_{opt1}$
p=0.05	$B^{R4JA}$	6	$L_{opt2}$
p=0.03	$B^{s,2}$	8	$L_{opt3}$
p=0.03	$B^{s,3}$	8	$L_{opt2}$
p=0.94	$B^{s,1}$	6	$L_{opt4}$
p=0.94	$B^{R4JA}$	6	$L_{opt2}$
p=0.98	$B^{s,2}$	8	$L_{opt5}$
p=0.98	$B^{s,3}$	8	$L_{opt6}$

## Data Availability

Not applicable.

## Abbreviations

The following abbreviations are used in this manuscript:

AWGN	Additive white Gaussian noise
BER	Bit error rate
BICM	Bit interleaved coded modulation
DM	Distribution matcher
JSCC	Joint source-channel coding
JSCCM	Joint source-channel coded modulation
PAS	Probabilistic amplitude shaping
PEG	Progressive edge growth
QAM	Quadrature amplitude modulation
